# Emergence and Phylodynamics of Influenza D Virus in Northeast China Reveal Sporadic Detection and Predominance of the D/Yamagata/2019 Lineage in Cattle

**DOI:** 10.3390/v18010093

**Published:** 2026-01-09

**Authors:** Hongjin Li, Weiwen Yan, Xinxin Liu, Bing Gao, Jiahuizi Peng, Feng Jiang, Qixun Cui, Che Song, Xianyuan Kong, Hongli Li, Tobias Stoeger, Abdul Wajid, Aleksandar Dodovski, Chao Gao, Maria Inge Lusida, Claro N. Mingala, Dmitry B. Andreychuk, Renfu Yin

**Affiliations:** 1 State Key Laboratory for Diagnosis and Treatment of Severe Zoonotic Infectious Diseases, Key Laboratory of Zoonosis Research, Ministry of Education, Department of Preventive Veterinary Medicine, College of Veterinary Medicine, Jilin University, Changchun 130062, China; 2 College of Food Science and Engineering, Jilin University, Changchun 130062, China; 3 College of Veterinary Medicine, Shanxi Agricultural University, Taiyuan 030801, China; 4 Institute of Lung Health and Immunity (LHI), Comprehensive Pneumology Center (CPC), Helmholtz Zentrum München, Member of the German Center for Lung Research (DZL), 85764 Munich, Germany; 5 Department of Biotechnology, Balochistan University of Information Technology, Engineering and Management Sciences, Quetta 87100, Pakistan; 6 Department for Avian Diseases, Faculty of Veterinary Medicine, Ss. Cyril and Methodius University in Skopje, Lazar Pop Trajkov 5-7, 1000 Skopje, North Macedonia; 7 Faculty of Medicine, Universitas Airlangga, Surabaya 60115, Indonesia; 8 Research Center on Global Emerging and Re-Emerging Infectious Diseases, Institute of Tropical Disease, Universitas Airlangga, Surabaya 60115, Indonesia; 9 Livestock Biotechnology Center, Philippine Carabao Center, Science City of Muñoz, Nueva Ecija 3120, Philippines; 10 Reference Laboratory for Avian Viral Diseases, FGBI “Federal Centre for Animal Health” (FGBI “ARRIAH”), Vladimir 600901, Russia

**Keywords:** influenza D virus, D/Yamagata/2019 lineage, cattle reservoir, phylogeography, China

## Abstract

Influenza D virus (IDV), an emerging orthomyxovirus with zoonotic potential, infects diverse hosts, causes respiratory disease, and remains poorly characterized in China despite its global expansion. From October 2023 to January 2025, we collected 563 nasal swabs from cattle across 28 farms in Jilin Province, Northeast China, and identified seven IDV-positive samples (1.2%), recovering two viable isolates (JL/YB2024 and JL/CC2024). Full-genome sequencing revealed complete, stable seven-segment genomes with high nucleotide identity (up to 99.9%) to contemporary Chinese D/Yamagata/2019 strains and no evidence of reassortment. Maximum-likelihood and time-resolved Bayesian phylogenies of 231 global hemagglutinin-esterase-fusion (HEF) sequences placed the Jilin isolates within the East Asian D/Yamagata/2019 clade and traced their most recent common ancestor to approximately 2017 (95% highest posterior density: 2016–2018), suggesting a cross-border introduction likely associated with regional cattle movement. No IDV was detected in parallel surveillance of swine, underscoring cattle as the principal reservoir and amplifying host. Bayesian skyline analysis demonstrated a marked decline in global IDV genetic diversity during 2020–2022, coinciding with livestock-movement restrictions imposed during the COVID-19 pandemic. Collectively, these findings indicate that IDV circulation in China is sporadic and geographically localized, dominated by the D/Yamagata/2019 lineage, and shaped by multiple independent incursions rather than a single emergence. Both the incorporation of IDV diagnostics into routine bovine respiratory disease surveillance and cattle-import quarantine programs, and the adoption of a One Health framework to monitor potential human spillover and future viral evolution, were recommend.

## 1. Introduction

**Figure 1:**
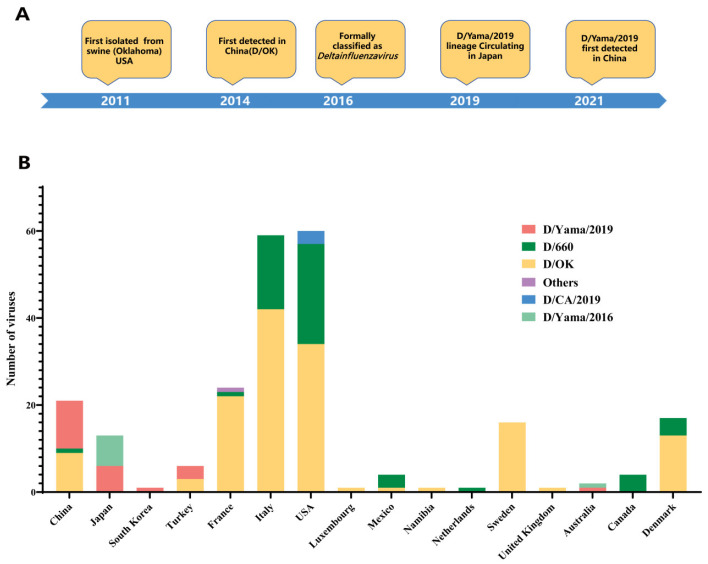
Historical timeline and global distribution of Influenza D Virus (IDV). (**A**) Timeline of key IDV historical events. (**B**) Global distribution of reported IDV isolates. This panel summarizes the worldwide occurrence of all lineages based on published sequence data and isolation records, with each country labeled by the number of reported isolates.

Influenza D virus (IDV), a recently identified member of the *Orthomyxoviridae* family, is most closely related to influenza C virus but is antigenically distinct, showing no cross-reactivity in hemagglutination-inhibition assays [1]. First isolated in 2011 from pigs with respiratory illness in Oklahoma, USA, IDV was formally classified as a new influenza genus, *Deltainfluenzavirus*, in 2016 [2] (Figure 1A). Subsequent research established cattle as the primary natural reservoir, with IDV recognized as an important contributor to the bovine respiratory disease (BRD) complex [3]. While IDV infection in cattle typically causes mild to moderate respiratory symptoms, co-infecting with other pathogens can lead to BRD, leading to significant economic losses in the cattle industry.

IDV exhibits a broad host range, infecting not only cattle and swine but also sheep, goats, camelids, and wild ungulates such as buffalo, giraffe, and wildebeest [3,4,5,6]. Serological surveys indicate a potential zoonotic risk, with a notable proportion of individuals in close contact with cattle- such as farmers and veterinarians—showing detectable antibodies against IDV, despite the absence of confirmed human cases to date [7,8]. In vitro studies demonstrate IDV can replicate in human respiratory epithelial cells, and animal model experiments in ferrets and guinea pigs show efficient transmission, raising concerns about the virus could cross the species barrier under favorable conditions [1,6,9]. These findings underscore the need for ongoing surveillance of IDV as a possible zoonotic pathogen.

Genetic diversity in IDV is driven primarily by variations in the hemagglutinin-esterase fusion (HEF) gene, which encodes the surface glycoprotein responsible for receptor binding and is the major antigenic target [10]. Based on HEF gene sequences, IDV is currently classified into six major lineages: D/OK (the prototype lineage first identified in Oklahoma, USA), D/660 (initially detected in North America and Europe), D/Yamagata/2016 and D/Yamagata/2019 (both first identified in Japan), D/CA2019 (a reassortant lineage discovered in California, USA) and D/France [1,11,12,13]. Among the identified IDV lineages, the D/OK and D/660 lineages are the most prevalent globally, demonstrating widespread circulation. In contrast, the D/CA2019, D/France, D/Yamagata/2016, and D/Yamagata/2019 lineages were previously regarded as geographically restricted [14,15] (Figure 1B). In recent years, some geographically restricted lineages, such as D/Yamagata/2019, have expanded across the globe, raising questions about their origin, dissemination routes, and ecological dynamics.

Despite increasing reports of IDV worldwide, data from China remain limited, and the virus’s phylogeographic history in the region is poorly defined. Northeastern China, characterized by intensive cattle production and active cross-border livestock trade with Russia and the Korean Peninsula, represents a critical yet under-sampled region for understanding IDV circulation. Here, we conducted molecular detection, full-genome sequencing, and comprehensive phylogenetic and phylodynamic analyses of IDV isolates collected from cattle in Jilin Province between 2023 and 2025. Our objectives were to (i) determine the prevalence and genetic characteristics of circulating IDV strains, (ii) reconstruct the timing and routes of introduction into China, and (iii) evaluate the evolutionary dynamics and potential zoonotic implications of the dominant D/Yamagata/2019 lineage. These findings provide crucial insight into the emergence and ongoing evolution of IDV in East Asia and inform surveillance strategies within a One-Health framework.

## 2. Material and Methods

### 2.1. Sample Collection

**Figure 2:**
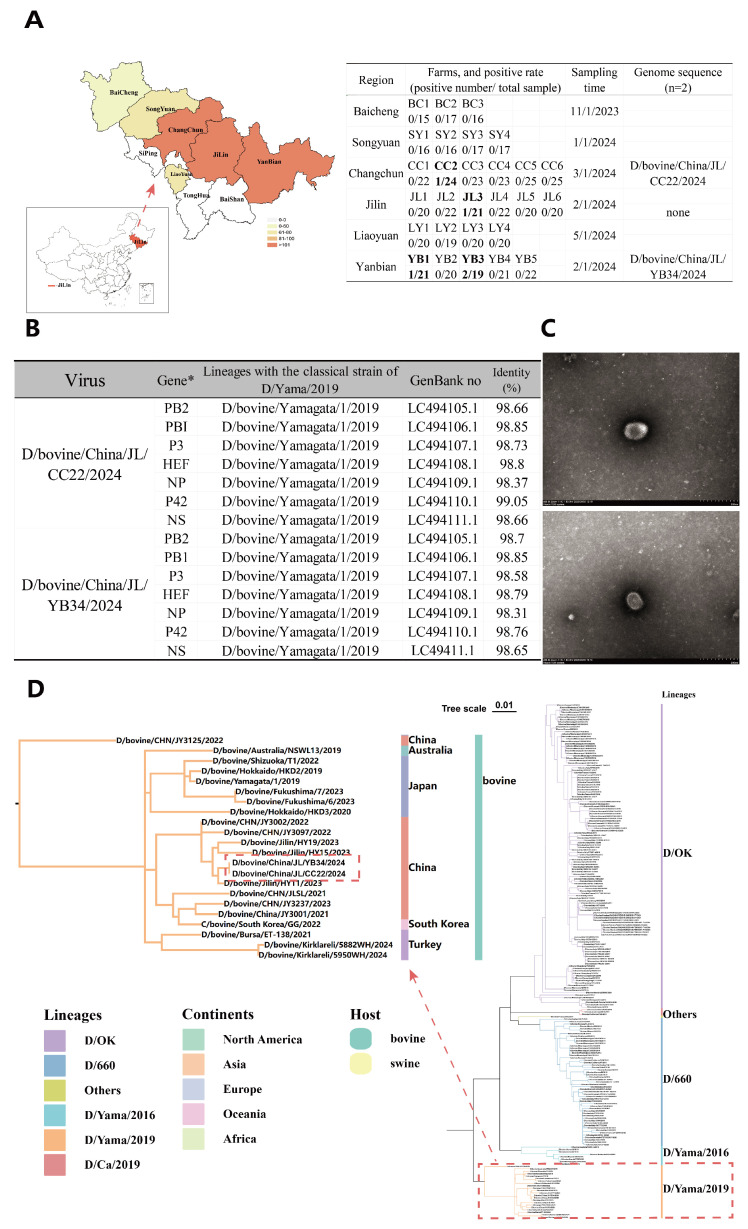
Geographic distribution, electron microscopy and phylogenetic analysis of IDV in China from 2023 to 2025. (**A**) Metadata for clinical samples and IDV isolates from cattle. Sampling sites and sample numbers are shown on the left map, while the identifiers of the 28 farms are listed in the table on the right. Farms with IDV-positive samples are highlighted in bold. (**B**) Genome comparison of the D/Yamagata/2019 reference strain with Jilin isolates D/bovine/China/JL/CC22/2024 and D/bovine/China/JL/YB34/2024. (**C**) Negative-stain electron microscopy of D/bovine/China/JL/CC22/2024 and D/bovine/China/JL/YB34/2024. Magnification = ×40.0k; Accelerating Voltage = 80.0 kV; Scale bar = 100 nm. (**D**) Phylogenetic and Bayesian phylodynamic analysis. HEF gene sequence and associated metadata were retrieved from the National Center for Biotechnology Information (NCBI) in November 2025, including complete HEF genes with epidemiological details (collection date, location, host species) (Table S1). Multiple sequence alignments were conducted using MAFFT in the PhyloSuite. The best-fitting model (GTR + Empirical + G4) was selected based on Bayesian Information Criteria (BIC) using Modelfinder in PhyloSuite. A maximum-likelihood phylogenetic tree was generated in IQ TREE with 10,000 ultrafast bootstrap replicates, using the D/OK (GenBank accession JQ922308) as the outgroup.

From October 2023 to January 2025, a total of 563 nasopharyngeal swabs were collected from clinically healthy cattle at the time of sampling on 28 farms, each housing at least 50 adult cows under intensive nutritional management, across in multiple regions of Jilin Province, Northeast China. Sampling sites were selected based on geographical location and breeding density and included Baicheng, Songyuan, Changchun, Jilin, Liaoyuan, and Yanbian (Figure 2A). At least three farms were randomly selected from each region, and the required sample size was calculated using the OpenEpi online tool (http://www.openepi.com/, accessed on 2 December 2025) based on the formula described by Fleiss et al. (2003) 16, assuming a confidence level of 99.99% and an acceptable margin of error (E) of 5%. The calculated minimum sample size was 560 animals; therefore, the final sampling of 563 cattle from 28 farms met and slightly exceeded this requirement (Figure 2A). The sampled herds consisted primarily of Yanbian Yellow cattle, Simmental beef cattle, and a small number of Holstein dairy cattle. Samples were performed under sterile conditions using flocked swabs placed in 10 mL EP tubes containing 5 mL of viral transport medium (40% glycerol, 2000 U/mL penicillin, 2 mg/mL streptomycin, 50 µg/mL gentamycin, 50 U/mL nystatin, and 0.5% bovine serum albumin). During field collection, samples were kept on dry ice and stored at −80 °C until processing, with strict cold-chain conditions maintained throughout transport and storage. Metadata including breed, sex, sampling date, and geographic location were recorded for each animal.

In parallel, a total of 734 achieved nasopharyngeal swabs from clinically healthy swine, collected between 2019 and 2022, across multiple Chinese provinces, including Jilin (*n* = 178), Heilongjiang (*n* = 139), Liaoning (*n* = 157), Inner Mongolia (*n* = 162), and Hebei (*n* = 98), were also tested for the presence of IDV.

### 2.2. Detection of IDV RNA

Concentration of viral particles prior to RNA extraction has been shown to improve detection efficiency and reduce false-negative rates in respiratory virus surveillance, particularly in nasopharyngeal swab samples containing low viral loads or high levels of host-derived background material [17,18,19]. Accordingly, viral enrichment was employed in this study to enhance the sensitivity of IDV RNA detection. For viral enrichment, 0.1 g polyethylene glycol 6000 and 0.02 g sodium chloride were added to 0.4 mL of transport medium containing the nasopharyngeal swabs, mixed thoroughly, and centrifuged and 12,000× *g* for 120 min at 4 °C. The supernatant was discarded and the pellet resuspended in 0.2 mL of nuclease-free water for RNA extraction using TRIzol Reagent (Sigma, Shanghai, China) [20]. The cDNA was generated by using the reverse transcription kit (Novoprotein, Suzhou, China), according to the manufacturer’s instructions. IDV RNA were detected by real-time RT-qPCR using a SYBR Green–based assay (FastStart Universal SYBR Green Master, Roche, Shanghai, China) targeting a 136 bp fragment of the PB1 gene. The primers were as follows: Forward, 5′-AAAATTCATTGCTGTTTGCA-3′; and Reverse, 5′-GTAATTCCAAAGCTATGTTTG-3′. Thermal cycling conditions were 95 °C for 3 min, followed by 40 cycles of 95 °C for 1 s and 60 °C for 10 s. The threshold line was manually set during the linear phase of exponential amplification above the baseline fluorescence, with ΔRn defined as 0.1. Samples with a Ct value ≤ 35 and a characteristic sigmoidal amplification curve were considered positive, where samples with Ct value > 35 or lacking specific amplification were considered negative. Positive samples were further tested using a universal RT-PCR assay amplifying a 733 bp fragment of the P42 region [21,22]. Amplicons were Sanger-sequenced, and only samples with confirmed sequence identity were deemed IDV-positive.

### 2.3. Viral Isolation and Whole-Genome Sequencing

Virus isolation was attempted for all IDV RNA-positive samples using Madin–Darby canine kidney (MDCK) cells. Cells were seeded in T-25 flasks overnight and inoculated with 100 µL of clarified swab supernatant from IDV RNA-positive specimens. After 1 h absorption at 37 °C, the inoculum was replaced with fresh maintenance medium (MEM supplemented with 2% FBS, 1% penicillin-streptomycin, 1% TPCK trypsin, 1% (200 mM) L-glutamine). Cytopathic effects (CPE) were monitored daily. Supernatant was collected 96 h post-challenge for IDV detection via RT-PCR targeting a 733 bp fragment of the P42 region, hemagglutination (HA) assays, and electron microscopy [21,22]. Following three consecutive passages, virus isolation was successfully from the positive samples.

The full-length genome was amplified using 7 pairs of primers in 20 μL reactions containing 10 μL 2× One Step Mix (Dye Plus), 1 μL enzyme mix, 1 μL each primer, 2 μL RNA template, and 5 μL RNase-free water. Cycling conditions were: 50 °C for 30 min, 94 °C for 3 min; 30 cycles of 94 °C for 30 s, 55 °C for 30 s, and 72 °C for 3 min; and a final extension at 72 °C for 7 min. Amplification products were analyzed by 1% agarose gel electrophoresis. Positive PCR products were purified using the Biospin gel extraction kit (Biospin, Beijing, China) and submitted to Jilin Kumei Biotechnology Co., Ltd. (Changchun, China) for Sanger sequencing.

### 2.4. Electron Microscopy

MDCK cells were infected with the IDV at a multiplicity of infection (MOI) of 2. At 96 h post-infection, the culture supernatants were collected, negatively stained and then examined by Hitachi HT-7800 electron microscope (Hitachi High-Tech, Mentor, OH, USA).

### 2.5. Phylogenetic and Bayesian Phylodynamic Analysis

HEF gene sequence and associated metadata for the IDV were retrieved from the National Center for Biotechnology Information (NCBI) in November 2025. The dataset comprised sequences with complete HEF genes and corresponding epidemiological information (collection date, location, and host species). Sequences with less than 85% gene coverage or incomplete metadata, such as missing collection dates, were excluded. To minimize potential phylogenetic bias caused by overrepresentation of highly similar sequences in public databases, sequence deduplication was performed using BioAider software v1.727. Nucleotide sequences were clustered with a similarity threshold of 99.9% (parameter: -c 0.999), and within each cluster, a single representative sequence was retained. This procedure reduced the dataset to 231 non-redundant HEF sequences, each annotated with relevant temporal, spatial, and host information (Table S1). Both reference strains and isolated from this study were retained for analyses. Multiple sequence alignments were conducted using MAFFT [23] within the PhyloSuite software v1.2.3. The best-fitting model (GTR + Empirical + G4) was selected based on Bayesian Information Criteria (BIC) using Modelfinder [24] in PhyloSuite [25]. A maximum-likelihood phylogenetic tree was generated in IQ TREE with 10,000 ultrafast bootstrap replicates [26], using the D/OK (GenBank accession JQ922308) as the outgroup.

For global discrete phylogenetic analysis, Bayesian phylogenetic dynamics analysis was executed with BEAST v1.10.4 [27] using a strict molecular clock and a Bayesian skyline coalescent prior. Markov Chain Monte Carlo (MCMC) chains were run for 500 million iterations, sampling every 50,000 steps. Convergence and effective sample sizes (ESS ≥ 200) were assessed with Tracer v1.7.1. The first 10% of states were discarded as burn-in. Maximum clade credibility (MCC) trees were generated with TreeAnnotator v1.10.4 [28,29,30,31,32] and visualized in FigTree v1.4.4. Geographic diffusion was subsequently analyzed and visualized with SpreaD3 [33].

## 3. Results

### 3.1. Sporadic Detection and Isolation of Influenza D Virus in Cattle from Northeast China

From October 2023 to January 2025, a total of 563 nasal swab samples were collected from cattle on 28 farms in Jilin Province, Northeast China. The herds consisted primarily of Yanbian Yellow cattle, Simmental beef cattle, and a small number of Holstein dairy cattle. All samples were screened for IDV using reverse transcription quantitative PCR (RT-qPCR) targeting the P42 gene. Seven samples (1.2%, 7/563; 95% CI: 0.6–2.5%) tested positive for IDV, detected on 4 of the 28 farms (14.3%; 95% CI: 4.0–32.7%) (Figure 2A). Geographically, IDV-positive cases were clustered within Jilin, with slightly higher detection rates in the Yanbian region compared with other surveyed areas. No IDV was detected in Holstein dairy cattle herds. Furthermore, IDV was not detected in any achieved swine samples (0/734) from multiple Chinese provinces analyzed in our laboratory. These findings suggest a low prevalence of IDV in Northeast Chinese cattle, with sporadic cases concentrated in specific localities.

Virus isolation was attempted from all IDV RNA-positive specimens with Ct values ≤ 35, each confirmed by sequencing a 733 bp fragment of the P42 region. Two IDV Jilin isolates were successfully recovered in MDCK cell culture: strain JL/YB2024 from a Yanbian Yellow cow in Yanbian and strain JL/CC2024 from a Simmental cow in Changchun, with corresponding Ct values of 32.3 and 29.1, respectively. The hemagglutinin (HA) titer ranged from 32 to 64, and RT-PCR sequencing results were consistent with the target sequences. MDCK cells were infected with the IDV at a multiplicity of infection (MOI) of 2. At 60 h post-infection (hpi), culture supernatants were harvested for the negative-staining electron microscopy. Transmission electron microscopy of the IDV particles in the supernatant revealed typical influenza virus morphology, with diameters ranging from approximately 100 to 120 nm. The particles exhibited a pleomorphic envelope and were predominantly spherical. Their surfaces were covered with densely packed spike-like protrusions, measuring 10–13 nm in length and 4–6 nm in diameter (Figure 2C).

### 3.2. IDV Jilin Isolates Exhibit No Reassortment and Cluster Within the D/Yamagata/2019 Lineage Now Predominant in China

Full-genome sequencing yielded complete coding sequences for all segments of the Jilin isolates D/bovine/China/JL/YB34/2024 (JL/YB2024) and D/bovine/China/JL/CC22/2024 (JL/CC2024) (GenBank PX427500 to PX427513). Both isolates exhibited the characteristic IDV genome structure, consisting of seven negative-sense RNA segments encoding the polymerase subunits (PB2, PB1, P3), hemagglutinin-esterase-fusion glycoprotein (HEF), nucleoprotein (NP), matrix protein (M; also known as P42), and the non-structural proteins NS1 and NS2, the latter produced by splicing of the NS segment.

Comparative analysis of IDV sequences available in the GenBank database showed that isolates JL/YB2024 and JL/CC2024 share 87.1–99.85% nucleotide identity across all coding segments with other IDV strains, exhibiting the highest similarity (99.85%) to strain HY11 (GenBank: OR880337) and JY3002 (GenBank: OR685129) (Figure 2B). Segment lengths and predicted protein sizes were fully consistent with other D/Yamagata/2019 trains, and no gene deletions or insertions were detected, indicating a stable viral genome within this lineage.

To place the Jilin isolates in a global context, a phylogenetic analysis of the HEF gene was conducted. A maximum-likelihood tree constructed from 231 IDV HEF sequences worldwide—including all Chinese IDV sequences—confirmed six primary global lineages as of 2025: D/OK, D/660, D/France, D/CA2019, D/Yamagata/2016, and D/Yamagata/2019. Of the 21 Chinese IDV sequences in the database (~9% of the total), three of these four lineages are represented. Earlier Chinese isolates (2014–2018), primarily from Shandong and Guangdong, belonged exclusively to the D/OK lineage. Since 2021, however, the D/Yamagata/2019 lineage has been detected across multiple provinces (Guizhou, Xinjiang, Gansu, Liaoning, and Jilin), supplanting D/OK as the predominant lineage.

**Figure 3:**
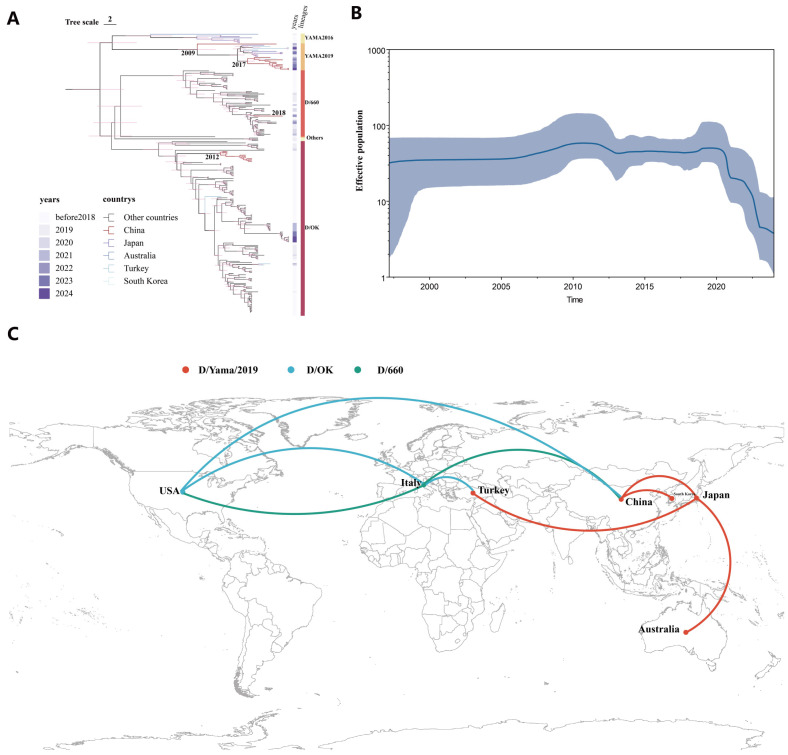
Phylogeography and Temporal Dynamics of IDV in China. (**A**) Maximum Clade Credibility (MCC) tree of IDV HEF gene. Global discrete phylogenetic analysis was executed using BEAST v1.10.4 with a strict molecular clock and a Bayesian skyline coalescent prior. Markov Chain Monte Carlo (MCMC) chains were run for 500 million iterations, sampling every 50,000 steps. Convergence and effective sample sizes (ESS ≥ 200) were assessed with Tracer v1.7.1 with the first 10% of states discarded as burn-in. MCC trees were generated with TreeAnnotator v1.10.4 and visualized in FigTree v1.4.4. Geographic diffusion was analyzed and visualized with SpreaD3. (**B**) Bayesian Skyline Plot of IDV Population Dynamics. (**C**) Global temporal dynamics of IDV lineages D/OK, D/6660, and D/Yamagata/2019 circulation.

The Jilin isolates clustered tightly within the Chinese D/Yamagata/2019 clade alongside other post-2021 Chinese strains and were closely related to contemporary isolates from Japan and South Korea, reflecting a shared lineage origin. Notably, a single 2023 isolate from Ningxia, north-central China, fell within the D/660 lineage—the first report of this lineage in Asia, previously identified only in Europe and North America. Excluding this D/660 case, the D/Yamagata/2019 lineage dominates accounts for 11 of the 21 known Chinese strains. Phylogenetic analysis of other genome segments confirmed that their internal genes of JL/YB2024 and JL/CC2024 also align with the D/Yamagata/2019 lineage, with no evidence of reassortment. Collectively, these findings indicate that the D/Yamagata/2019 lineage of IDV—once considered Japan-specific—has become endemic in Chinese cattle and has been the predominant IDV lineage in China since approximately 2021 (Figure 3A).

### 3.3. Phylogeography and Temporal Dynamics of Influenza D Virus in China

To investigate the timing and pathways of IDV spread, we conducted a time-resolved phylogeographic analysis of global IDV HEF sequences with discrete-trait mapping of geographic origins (East Asia vs. other regions). A Bayesian Markov chain Monte Carlo (MCMC) analysis generated a maximum clade credibility tree and evolutionary parameter estimates, elucidating the introduction history of the D/Yamagata/2019 lineage into China (Figure 3C).

Chinese D/Yamagata/2019-related sequences formed two distinct phylogenetic subclusters with separate origins. The primary cluster, including the Jilin isolates JL/YB2024 and JL/CC2024 in the study, most other Chinese D/Yamagata/2019 strains, and South Korean strains from 2021 to 2022, had an inferred time to the most recent common ancestor (tMRCA) in late 2017 (95% highest posterior density approximately 2016–2018). These findings suggest that the D/Yamagata/2019 lineage likely emerged in Asia during the late 2010s and was subsequently introduced into China. Although this pattern is consistent with, it does not definitively demonstrate, cross-border transmission from neighboring countries. This inference is further supported by the close genetic relationships observed among recent isolates from China, South Korea, and Japan; however, direct evidence identifying the precise geographic origin and transmission route of this lineage remains unavailable.

In contrast, a single outlier strain (D/bovine/CHN/JY3125/2022, GenBank OR685141) detected in China in 2023 belonged to a divergent D/Yamagata/2019 branch with a tMRCA around 2009, indicating an independent, earlier introduction that did not lead to widespread circulation. This strain appears either to have been a dead end or remained geographically localized and unsampled. Collectively, these findings suggest that the D/Yamagata/2019 lineage entered China through at least two separate events—around 2009 and again around 2017—with the latter introduction driving the current dominance of this lineage in Chinese cattle.

For comparison, estimated introduction times of other IDV lineages in China showed that the D/OK lineage likely entered around 2012, aligning with the first reported Chinese IDV cases in 2014. The single Chinese D/660 isolate (Ningxia, 2023, GenBank OR685153) belonged to a lineage with a tMRCA around 2018, suggesting a late-2010s introduction. None of the IDV lineages (D/OK, D/660, or D/Yamagata/2016) show evidence of widespread dissemination across China; each remains confined to specific regions or herds, indicating multiple localized introductions without a nationwide epidemic.

A Bayesian skyline plot of IDV genetic diversity suggested a stable global effective population size until 2020, followed by a sharp decline through 2021–2022 and a low in 2023 (Figure 3B). This reduction likely reflects decreased IDV circulation during the COVID-19 pandemic, when enhanced biosecurity and livestock-movement disrupted transmission chains and created a population bottleneck [34]. Despite the easing of restrictions in 2022–2023, IDV genetic diversity has not yet rebounded. The dominance of the D/Yamagata/2019 lineage in East Asia and the absence of new lineages post-2019 may further contribute to this decline. Continued surveillance will determine whether IDV regains genetic diversity or if certain lineages have been lost. Overall, this phylodynamic analysis underscores the impact of external factors, such as pandemic-related restrictions, on IDV spread and provides a timeline for the introductions of key lineages into China.

## 4. Discussion

This study provides the comprehensive molecular characterization, phylogeography analysis, and temporal dynamics of the D/Yamagata/2019 lineage of influenza D virus in China. IDV was detected sporadically (1.2%) among cattle in northeast China- a prevalence lower than that reported in other Chinese cattle studies [22,35]. Two Jilin isolates (designated JL/YB2024 and JL/CC2024, from Yanbian Yellow cattle and Simmental cattle, respectively) were successfully recovered in cell culture, exhibiting HA titers of between 32 and 64. Negative-staining election microscopy confirmed the isolation of infectious IDV (Figure 2C).

The complete coding sequences of all genome segments from the two Jilin isolates was obtained through full-genome sequencing. Comparison with IDV sequences in the GenBank database suggested no gene deletions or insertions. Across all coding segments, the Jilin isolates showed high nucleotide identity to other IDV strains, with the highest similarity (98.8%) to viruses within the D/Yamagata/2019 lineage. Although multiple linages circulate globally, the phylogenetic analysis suggest that the Jilin isolates share a recent ancestor with D/Yamagata/2019 strains and represent a predominant lineage currently circulating in China.

Despite the virus’s swine origin, parallel surveillance of swine farms in the region detected no IDV infections, suggesting a lack of sustained transmission in pigs [36,37]. In most cases, the absence of IDV in pigs, despite its presence in nearby cattle, indicates a host barrier limits interspecies transmission. Although pigs can be experimentally infected and sporadic field cases have been reported, and although IDV could be potentially be transmitted from bovines to pigs and adapt through specific mutations that enhance intraspecies transmission, the virus has not become endemic in swine herds, unlike swine influenza A viruses [37,38]. Furthermore, IDV was not detected (0/734) in any achieved swine samples from multiple Chinese provinces analyzed in our laboratory, consistent with previous reports of Vietnamese and Chinese swine farms [36,37]. Our finding, together with these earlier studies, reinforce that cattle are the primary reservoir and amplifying host for IDV in the region, with pigs playing only a negligible epidemiological role. This underscores the need to prioritize bovine-focused surveillance and control measures to manage IDV spread effectively.

Phylogenetic and phylodynamic analyses indicate that the D/Yamagata/2019 lineage entered China through at least two independent events. The dominant introduction occurred around 2017 (tMRCA: 2016–2018) and led to widespread circulation across provinces such as Jilin, Guizhou, Xinjiang, Gansu, and Liaoning, likely facilitated by regional cattle trade or movement, as evidenced by its close genetic similarity to a 2022 South Korean strain D/bovine/South Korea/GG/2022 (GenBank OQ946987). An earlier introduction around 2009, represented by strain CHN/JY3125/2022 (GenBank OR685141), appears to have been a dead-end event, with no subsequent detections, suggesting it either failed to establish or remained geographically limited. These findings highlight a complex introduction history for the D/Yamagata/2019 lineage in China, driven by multiple incursions rather than a single emergence. Despite its broad geographic reach, the lineage’s spread remains patchy and localized, consistent with IDV’s dependence on farm-to-farm transmission and restricted animal movement [39,40].

The absence of widespread IDV outbreaks across China or significant interspecies transmission further distinguishes IDV from highly transmissible viruses such as influenza A in swine or poultry [41]. The D/OK lineage, prevalent in the 2010s, and the single D/660 isolate detected in Ningxia in 2023 remained geographically restricted, while the D/Yamagata/2019 lineage, though currently dominant, is primarily bovine-specific. IDV transmission likely occurs via direct contact or short-range aerosols in farm settings, sustaining localized outbreaks but rarely spreading long distances without animal movement. Cattle importation and regional livestock trade appear to be key drivers of IDV introductions, a view supported by the observed decline in viral genetic diversity during the COVID-19 pandemic (2020–2021), when trade and movement restrictions likely disrupted transmission chains [42]. Strengthening biosecurity measures and health screening for imported cattle could help mitigate future IDV incursions.

Though the zoonotic potential of IDV has not yet been clearly established and no direct evidence was obtained in the present study, it nonetheless warrants careful attention in further investigations. Similarly to ICV, a common cold virus associated with mild upper respiratory tract infections in humans, IDV can bind to human airway receptors, specifically 9-*O*-acetyl-*N*-acetylneuraminic acid (9-*O*-Ac-Neu5Ac), which are present on human respiratory cells, thereby enabling viral attachment and replication [6,43]. Moreover, IDV is capable of replicating in well-established mammalian models commonly used for AIV studies, such as ferrets and guinea pigs, and has been shown to cause respiratory disease in serval mammalian species, including cattle [44,45]. Although no confirmed cases of clinical disease caused by IDV in humans have been reported to date, multiple seroepidemiological studies have demonstrated IDV-specific seropositivity in human populations, particularly among individuals with occupational exposure to cattle [7,8,46,47]. These findings indicate that humans can be exposed to IDV and mount a specific antibody response, suggesting the occurrence of subclinical or asymptomatic infections [3,48]. The detection of IDV-specific antibodies in humans, together with the virus’s ability to bind human airway receptors and replicate in mammalian models, underscores its potential zoonotic risk and highlights the need for continued surveillance at the animal–human interface.

In conclusion, this study suggests that IDV is detected sporadically in cattle in Northeast China and that the D/Yamagata/2019 lineage represents the predominant lineage currently circulating, likely introduced via cross-border cattle movement from neighboring countries. Cattle appear to be the primary reservoir, with no evidence of sustained transmission in swine. To mitigate IDV spread, we recommend incorporating IDV testing into routine veterinary surveillance for bovine respiratory diseases and into quarantine protocols for imported cattle. Continuous monitoring for genetic changes, including reassortment or host-range adaptations, is also critical. A One Health approach, addressing both animal circulation and potential human health risks, is essential. By documenting the dominance of the D/Yamagata/2019 lineage in Northeast of Chinese cattle, this study provides a foundation for ongoing vigilance at the animal–human interface.

## Data Availability

The data presented in this study is available on request from the corresponding author.

## Supplementary Materials

The following supporting information can be downloaded at: https://www.mdpi.com/article/10.3390/v18010093/s1. Table S1: Metadata for 231 non-redundant IDV HEF sequences used in phylogenetic analysis, including collection year, location, host, strain designation, GenBank accession numbers, and sequences.
